# Recent Progress on Lipid Intake and Chronic Kidney Disease

**DOI:** 10.1155/2020/3680397

**Published:** 2020-04-24

**Authors:** Ke Pei, Ting Gui, Chao Li, Qian Zhang, Huichao Feng, Yunlun Li, Jibiao Wu, Zhibo Gai

**Affiliations:** ^1^College of Traditional Chinese Medicine, Shandong University of Traditional Chinese Medicine, Jinan 250355, China; ^2^Key Laboratory of Traditional Chinese Medicine for Classical Theory, Ministry of Education, Shandong University of Traditional Chinese Medicine, Jinan 250355, China; ^3^First Clinical Medical College, Shandong University of Traditional Chinese Medicine, Jinan 250355, China; ^4^Acupuncture and Massage College, Shandong University of Traditional Chinese Medicine, Jinan 250355, China; ^5^The Affiliated Hospital of Shandong University of Traditional Chinese Medicine, Jinan 250011, China; ^6^Department of Clinical Pharmacology and Toxicology, University Hospital Zurich, University of Zurich, 8006 Zurich, Switzerland

## Abstract

The incidence of chronic kidney disease (CKD) is associated with major abnormalities in circulating lipoproteins and renal lipid metabolism. This article elaborates on the mechanisms of CKD and lipid uptake abnormalities. The viewpoint we supported is that lipid abnormalities directly cause CKD, resulting in forming a vicious cycle. On the theoretical and experiment fronts, this inference has been verified by elaborately elucidating the role of lipid intake and accumulation as well as their influences on CKD. Taken together, these findings suggest that further understanding of lipid metabolism in CKD may lead to novel therapeutic approaches.

## 1. Introduction

Lipids contain many molecules that contribute to structural components of membranes and signal transduction that regulates a variety of cellular events to maintain physiological homeostasis. Recent research on the relationship between lipid disorders and kidney disease concluded that when the balance of lipid uptake, synthesis, and excretion in the kidney is disrupted, lipid accumulation occurs and causes nephrotoxicity and chronic kidney disease (CKD) [1]. Chronic kidney disease represents a serious public health problem due to its increased morbidity and prevalence worldwide. Dyslipidemia is frequently found in every stage of CKD, and lipid disorders aggravate the progression of CKD. In fact, dyslipidemia leads to impairment of the glomerular filtration barrier and proteinuria. The increase in serum triglyceride to high-density lipoprotein (HDL) ratio is a characteristic of dyslipidemia in CKD patients and is also an independent indicator of disease progression. Several clinical studies have confirmed that an elevated serum triglyceride to HDL ratio has a major impact on the decrease of the estimated glomerular filtration rate (eGFR) and the development of CKD [2]. Dyslipidemia itself is not enough to cause kidney injury; however, it is one of the necessary components of the multistep mechanisms since it also induces inflammation, oxidative stress, and lipotoxicity [1]. CKD also leads to marked alterations of secondary abnormalities in lipid metabolism [3]. Several studies have documented that CKD leads to decreased fatty acid oxidation (FAO), which could be an additional mechanism resulting in lipid accumulation [4]. An increased abundance of saturated C16 or C20 free fatty acids (FFAs) accompanied by impaired *β*-oxidation has been noted in the late stage of CKD, contributing to further accumulation of saturated fatty acids (SFA) and leading to cell dysfunction, cell death, and the further progression of CKD [5]. Nowadays, most studies emphasize the impact of lipid metabolism disorders in chronic kidney disease (CKD). Less attention has been paid to the lipid intake of patients with CKD and the possibility of using this as a tool to improve CKD. In conclusion, this article focuses on the mechanism of lipid intake leading to CKD and possible therapeutic approaches.

## 2. High-Fat Diet Intake and CKD

A high-energy, high-fat diet (HFD), especially one with a high-SFA intake, promotes obesity and metabolic syndrome abnormalities [6–10]. The excessive intake of energy, including a HFD, causes an imbalance between renal lipogenesis and lipolysis, which is considered to be an initial reason for renal lipid accumulation, and eventually renal injury [11]. After evaluating the population-based dietary pattern with the risk of incident CKD in a 6.1-year follow-up, it turned out that a high-fat, high-sugar diet was associated with an observable increase in the occurrence (46%) of incident CKD, whereas a lactovegetarian diet might be protective against the incidence of CKD by 43% [12]. Western diet patterns characterized by red and processed meat, saturated fat, and sweets were positively associated with a decrease in renal function after 11 years of follow-up in the participants from the Nurses' Health Study (NHS). Furthermore, participants who were in the highest third of the high-fat, high-sugar dietary pattern had a 49% increased rate of developing CKD, independent of diabetes and hypertension [13]. These studies show that dietary intake is a vital modifiable risk factor that is associated with delaying or preventing the development of CKD in humans [14–16]. However, there were also clinical studies showing a reverse association of body mass index (BMI) and survival in patients with advanced chronic kidney disease (CKD) as compared to the general population [17, 18]. Adequate management with respect to the specific role of obesity in different stages of CKD should be further integrated in routine renal care [19].

## 3. Lipid Uptake in the Kidney

Renal cells take up lipoproteins by scavenger receptors (SRs), including SR-B (CD36), SR-A, and SR-E (LOX-1). Under pathogenic stimulation, the scavenger receptors are dysregulated and their level is positively associated with the level of renal injury [20]. Furthermore, the expression of scavenger receptors (SRs) is not downregulated by intracellular cholesterol. As a result, cells expressing SRs can internalise a mass of cholesterol esters, leading to foam cell formation. All of these mechanisms result in the excessive absorption of lipids by kidney cells [1].

### 3.1. CD36-Mediated Lipid Uptake

**Figure 1:**
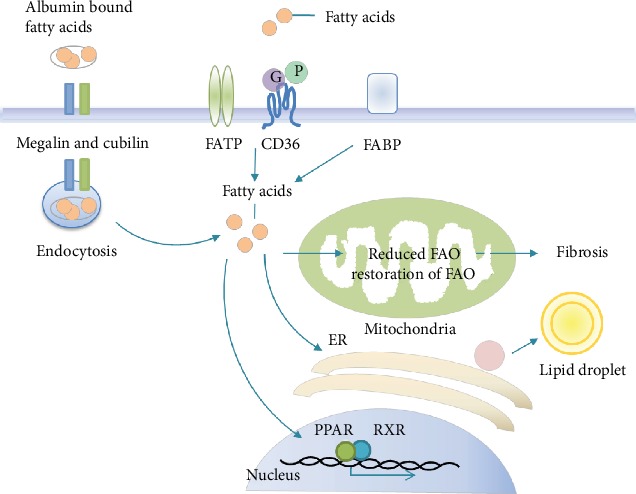
Schematic representation of fatty acid cellular uptake in the kidneys. FA transport across the plasma membrane occurs mainly by protein-mediated mechanisms or receptor-mediated endocytosis. In the cells, FAs bind to different FABPs with respect to the subcellular localization and have multiple functions in energy generation and storage, membrane synthesis, and activation of nuclear transcription factors like PPAR/RXR. Abbreviations: FATP: fatty acid transport protein; FABP: fatty acid-binding protein; FAO: fatty acid oxidation; PPAR: peroxisome proliferator-activated receptor; RXR: retinoid X receptor.

CD36 is expressed on various cell types, such as monocytes, macrophages [21], and proximal tubular cells (PTCs) [22], and mediates phagocytosis and degradation of oxidized low-density lipoprotein (ox-LDL) [23]. CD36 is also one of the most important transporters and transmembrane glycoproteins with a high-affinity uptake of long-chain fatty acid (LCFA, as schematically shown in Figure 1) [24–29]. Its level is positively correlated with the degree of renal injury if pathogenic stimuli, such as aminonucleoside (PA), have been applied [20]. Most nonesterified fatty acids (NEFAs) in the blood are combined with carrier proteins (mostly albumin), and their uptake requires dissociation from carrier proteins mediated by CD36 [30]. Albumin-bound fatty acids are extracted from filtrate by albumin endocytosis mediated by CD36 or by a receptor in the proximal renal tubules [31]. Although the filtration barrier prevents exposure of the proximal tubules to large lipoproteins, in individuals with proteinuria nephropathy including diabetic kidney disease (DKD), the proximal tubular cells are exposed to high amounts of filtered albumin and NEFAs. The excessive accumulation of nonesterified FFAs and triglycerides in the kidney leads to cytotoxicity, contributing to CKD progression [32]. The role of CD36 in the kidney has been investigated extensively [33]. SFA, which increase CD36 expression in podocytes, are major ligands for CD36, resulting in enhancement of FFA uptake, which will contribute to the generation of the vicious cycle [34]. Recently, the transgenic overexpression of CD36 in the kidneys of mice has been shown to induce lipid accumulation in the kidney [35]. It has been verified that CD36 increased podocyte apoptosis in primary nephrotic syndrome [36]. Saturated FFAs induce podocyte apoptosis through the CD36 signaling pathway by increasing oxidative stress [37]. CD36 has been reported to be upregulated in the kidney tissue of a nephrotic mouse. When glucose or fatty acid levels rise, the upregulation of CD36 can promote epithelial-mesenchymal transition in renal tubular epithelial cells and apoptosis of podocytes, thereby promoting the occurrence of diabetic nephropathy [36, 38]. Increased CD36 expression in the kidneys of CKD patients leads to renal dysfunction accompanied by systemic abnormalities, including proteinuria, renal lipid accumulation, and glomerular lesions [37, 39]. Recent studies have shown that renal abnormalities are attenuated in CD36^−/−^ mice, suggesting that CD36 plays an important role in the pathogenesis of kidney diseases [33, 37, 38]. The deletion of CD36 in mice largely reduced fatty acid uptake and ectopic renal lipid accumulation and prevented the progression of renal disease. In vitro, silencing CD36 almost abrogated inflammatory cytokine-induced fatty acid uptake, cellular FFA accumulation, and cellular stress. Ox-LDL uptake in renal tubular cells is mainly mediated by CD36 [22, 40]. A recent study found that the CD36-mediated signal pathway leads to proteinuria-induced tubulointerstitial injury [41, 42]. These researches suggest that CD36 can be a promising target for the treatment of renal injury [34].

### 3.2. Megalin- and Cubilin-Mediated Endocytosis

In addition, the renal proximal tubule retrieves albumin-bound FFA from the filtrate by megalin- and cubilin-mediated albumin endocytosis [31, 43–45]. It has been proposed that the excess of free fatty acids in the proximal tubule may result from the lipid cargo brought by increased filtered albumin, resulting in an increase in the rate of lipid uptake by albumin endocytosis through the receptor megalin and its extracellular binding partner cubulin [31, 43]. The megalin-cubulin complex is considered to be a low-affinity mechanism that operates in a high-capacity endocytic system that can readily account for the uptake of approximately 10 *μ*g/ml of albumin in the tubular filtrate [31]. Albumin is an efficient carrier of fatty acids. In megalin knockout mice fed with a high-fat diet, the kidney has lesser fatty acid-rich albumin uptake than the HFD-fed control mice [46]. Megalin could also mediate proximal tubular uptake of L-FABP, which may also exert nephrotoxic effects [47].

### 3.3. SLC27 A2- and SR-A-Mediated Lipid Uptake

In addition to CD36, SLC27A1-6 and fatty acid transporter proteins take up fatty acids by mediating their transmembrane movement and capturing NEFAs with CoA synthetases. SLC27 A2 (FATP2) is highly expressed in renal tubules according to the human protein profile. Therefore, it is an important candidate for mediating the uptake of fatty acids in the kidney [4]. In vitro microperfusion and in vitro experiments with NEFA-bound albumin at concentrations mimicking apical proximal tubule exposure during glomerular injury showed remarkably reduced NEFA absorption and palmitate-induced apoptosis in microperfused Slc27a2^−/−^ proximal tubules and Slc27a2^−/−^ or FATP2 shRNA-treated proximal tubule cell lines in comparison to wild-type or scrambled oligonucleotide-treated cells, respectively. Thus, FATP2 is a major apical proximal tubule NEFA transporter that regulates lipoapoptosis and may be a target that can prevent CKD progression [48]. Restoring PPARA signaling through drugs or genetics means to improve FAO or block FA transporter SLC27A2 and help protect mice from renal toxicity [49]. Scavenger receptor A1 (SR-A1), which is highly expressed in macrophages, is capable of taking up oxidized LDL (ox-LDL) and is not regulated by intracellular cholesterol [50–53]. Lipids become oxidized and bound to extracellular matrix proteins under conditions of inflammation. Inflammatory cytokines and growth factors enhance the expression of influx pathways, especially SR-A, and inhibit efflux pathways, resulting in a significant accumulation of intracellular lipids [3]. SR-A is also expressed in low levels on renal tubular epithelium. Both mRNA and protein levels of SR-A were increased in 5/6 nephrectomized CRF rats, which contribute to elevated levels of lipid accumulation in the remnant kidney [54]. Additionally, studies on hypercholesterolemic mice showed that an increase of SR-A interstitial cells suggest that SR-A+ may play a role in inflammation and renal fibrogenesis [22].

## 4. Ectopic Lipid Accumulation and Fatty Acid-Induced Renal Toxicity

**Figure 2:**
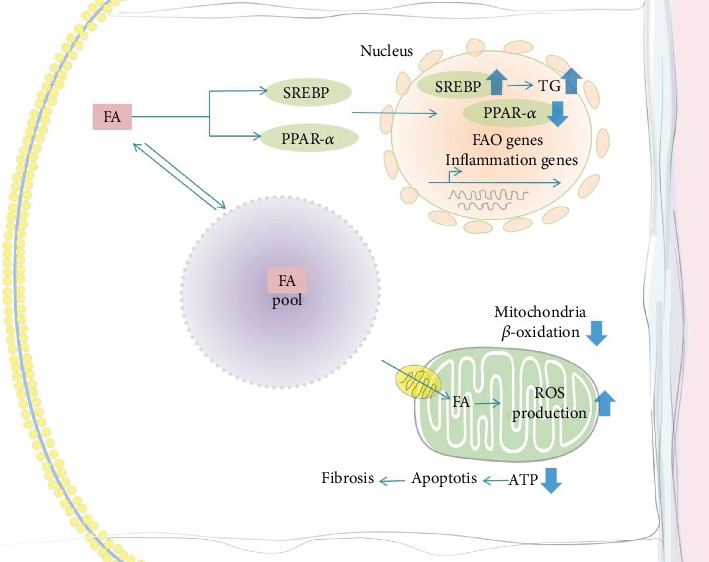
Ectopic lipid accumulation and fatty acid-induced renal toxicity. FA is stored in the global triglyceride pool or oxidized in mitochondria to produce ATP. FA, on the one hand, increases the expression of SREBP and, on the other hand decreases the activity of PPAR-*α*, both increasing the renal triglyceride level and proinflammatory cytokines. Accumulated FFAs are trapped in the mitochondrial matrix, resulting in mitochondrial reactive oxygen species (ROS) production and dysfunction. ATP depletion contributes to apoptosis, resulting in fibrosis. Blue arrows indicate what is being downregulated (down arrows) or upregulated (up arrows). Abbreviations: FA: fatty acid; SREBP: sterol regulatory element-binding protein-1c; PPAR-*α*: peroxisome proliferator-activated receptor-alpha; FAO: fatty acid oxidation; ROS: reactive oxygen species; ATP: adenosine triphosphate.

The toxicity of lipids includes fatty acid toxicity and cholesterol toxicity. In this paper, we focus on the relationship between fatty acid toxicity and CKD [55–58]. Ectopic lipid (renal lipid accumulation at ectopic sites), also known as lipotoxicity, refers to the accumulation of FAs in nonadipose tissue. Kidney biopsy specimens from patients with diabetic nephropathy showed lipid accumulation in the glomeruli and tubulointerstitium compared to the normal control group [59, 60]. One hypothesis is that NEFAs bound to serum albumin pass through the glomerular filtration barrier, promoting toxicity by converting NEFAs into toxic proinflammatory metabolites [61]. Since fatty acids are the preferred energy source for proximal tubular cells, a reduction in fatty acid oxidation in CKD affects renal lipid metabolism by disturbing the balance between fatty acid synthesis, uptake, and consumption [35]. As a result, increased intracellular lipid accumulation has a key role in the development of renal disease [35, 38, 62, 63]. Genes associated with FAO are downregulated [35] in the kidneys of mice and humans with CKD. Experiments have confirmed that lipids play a direct role in the initiation and progression of CKD [63–67]. More precisely, ample evidence has shown that ectopic lipids are associated with structural and functional changes in mesangial cells, podocytes, and proximal tubular epithelial cells in the kidneys to induce obesity-related CKD progression [68–70]. It has also been demonstrated that strategies to reduce lipid levels have beneficial effects on kidney health [71, 72] (Figure 2).

### 4.1. Lipid Accumulation and Glomerular Injury

Palmitate is the predominant circulating saturated FFA and is increased in states of insulin resistance [73]. In an animal model of type 1 diabetes, the increase in sterol regulatory element-binding protein (SREBP) in the renal cortex led to the upregulation of enzymes responsible for FFA synthesis, resulting in high triglyceride content [74]. In animals with a normal serum lipid level but with overexpression of SREBP-1, the renal triglyceride level was elevated and mesangial matrix expansion was increased with proteinuria and glomerulosclerosis [3, 74]. High CD36 expression is associated with an increased FFA uptake by podocytes, together with decreased *β*-oxidation and the accumulation of intracellular lipids. Accumulated FFAs become trapped in the mitochondrial matrix, resulting in mitochondrial reactive oxygen species (ROS) production, lipid peroxidation, and mitochondrial damage and dysfunction [75]. The unbalanced transport and oxidation of FFAs, with an impaired antioxidant response, impair podocyte structure, finally resulting in glomerulopathy [20]. In conclusion, increased triglyceride synthesis and FA uptake by mesangial cells induce diabetic glomerulopathy [3, 74, 76].

### 4.2. Lipid Accumulation and Renal Tubular Injury

Large lipid droplets accumulate in proximal tubular cells during the nephrotic syndrome called “lipid nephropathy.” Tubular NEFA uptake, as well as glomerular proteinuria, and plasma NEFA concentrations all increased in obesity. Excess NEFAs in albuminuria lead to tubulointerstitial injury [77, 78]. Triglyceride accumulation per se in proximal tubules also stimulates renal gluconeogenesis and increases tubular atrophy and interstitial fibrosis [32]. Toxic lipid metabolites alter the redox environment of cells to a more oxidized state, which then reduces their ability to oxidize NEFAs, resulting in further fat accumulation and insulin resistance [32]. Renal proximal tubule cells are the most energy-demanding cells in the body and oxidize fatty acids to produce ATP; therefore, renal tubular epithelial cells (TECs) critically depend on FAO as their energy source. The extraction of fatty acids from the human kidney is linearly related to the concentration of plasma fatty acids [79]. It has been found that when the expression of key enzymes and regulators of FAO is low, intracellular lipid deposition is high in tubulointerstitial fibrosis [80]. Inhibition of FAO in TECs causes ATP depletion, contributing to apoptosis, dedifferentiation, and intracellular lipid deposition, which induce fibrosis (see Figure 2 for details) [80]. In addition, excessive FAs might affect epithelial cells independently from FAO. Lipotoxicity exists and contributes to epithelial injury by directly activating apoptotic signaling or by indirectly promoting the infiltration of inflammatory cells, which is the main factor for fibrosis [81]. Increasing amounts of NEFAs bound to albumin impair mitochondrial respiration and peroxide-mediated apoptosis of tubular cells [82]. Purified and endotoxin-free albumin bound to palmitate and nonpurified albumin products had a similar influence on cultured tubule epithelial cells (TECs). It has been reported that albumin-bound FAs activate PPAR-*δ* and then raise tubular inflammation via proinflammatory metabolites in vivo [83]. Harris et al. confirmed these conclusions and demonstrated that an excess of palmitic acid induces endoplasmic reticulum (ER) stress in the kidney peritubular capillary (PTC) model [84]. Apoptosis and oxidative and ER stress form a proinflammatory environment around the renal PTC [85]. Overall, free long-chain nonesterified saturated fatty acids are toxic when added to cultured cells [4].

## 5. Therapy

### 5.1. Diet Therapy and Medication

Several studies have highlighted the effectiveness of dietary and lifestyle interventions and pharmacological strategies in kidney dysfunction [6, 7, 86–89]. Significant improvements in renal function through weight loss have suggested the reversibility upon early intervention, playing a role similar to early diabetic nephropathy [70]. Except for increased physical activity, the reduction of caloric intake is strongly recommended for overweight DKD patients [90, 91]. The negative impact of ox-LDL inducing apoptosis in human cultured podocytes can be effectively suppressed by statins in vitro [92]. Saturated FFAs in the pathogenesis of T2DM are thought to induce podocyte endoplasmic reticulum stress and apoptosis [93, 94]. Podocytes loss is a hallmark of DKD, and these cells are vulnerable to damage from saturated rather than monounsaturated FFAs [94]. Endoplasmic reticulum stress and podocyte cell death could be improved by inducing stearoyl-CoA desaturase [95], which converts saturated FFAs to monounsaturated FFAs and is upgraded in podocytes in biopsy specimens from patients with DKD [19]. Hence, monounsaturated fatty acids were beneficial to DKD. Several studies have demonstrated that circulating polyunsaturated fatty acids have beneficial effects on protecting renal function [96]. Long-chain polyunsaturated omega-3 fatty acids (n-3 PUFAs) (eicosapentaenoic acid (EPA) and docosahexaenoic acid (DHA)), which are obtained mainly from cold water fish, have diverse beneficial effects [97]. It has also been confirmed that renal function improved when individuals were given EPA+DHA at doses equal to two portions of fish per week [96]. N-3 and n-6 PUFAs were found to have a positive influence on DKD outcomes via the attenuation of endothelial dysfunction and inflammation as well as the improved control of dyslipidemia and hypertension [98]. High consumption of n-3 PUFAs and n-6 PUFAs was associated with a decrease and increase risk of CKD, respectively [15]. N-3 PUFAs may have therapeutic potential in ameliorating proteinuria in CKD and decreasing triglycerides and inflammation in dialysis patients. As part of a plant-based diet with low content of SFA, increasing consumption of oil-rich fish may benefit patients with CKD or that have the risk of developing CKD [99]. Furthermore, high n-6 PUFA or low SFA intake has been associated with an increased survival rate in dialysis patients [77, 78]. It has been proposed that improving the quality of dietary fat can ameliorate the clinical rick and outcome of CKD [79]. Of note, the ratio of n-3 : n-6 is more important than the PUFA intake and the low n-3 : n-6 ratio is detrimental for the health of human beings [80, 81]. The unbalanced n-6/n-3 PUFAs ratio, reaching up to 20 : 1 in some cases, can affect the onset of many underlying diseases, including CKD [82–84].

### 5.2. Targeted Therapy

The blocking of CD36-governed cellular processes is a promising strategy for treating obesity-related nephropathy. Several studies have demonstrated that metabolic dysfunction, fibrosis pathways, and proteinuria can be impeded by deficiency or blockade of CD36 [33, 82, 100, 101]. Blocking CD36 on podocytes in vitro resulted in cell function with reduced apoptosis and oxidative stress [37, 102–106]. Therefore, blocking the CD36-dependent pathway is expected to be a therapeutic strategy for a variety of kidney diseases, and novel CD36-targeting peptides have the ability of slowing the progression of CKD [107]. Considering the universal expression and cell-specific effects of CD36, future efforts should include the development of new peptides that target specific sites on the receptor and/or select cell populations to limit the potential for off-target effects and increase the efficacy of targeting CD36 in a variety of renal diseases [107]. Inhibition of the Renin-Angiotensin-Aldosterone System (RAAS) is the basis of the therapy for albuminuria and glomerular filtration. Up until now, very limited clinical trials have been carried out on RAAS inhibitors that primarily target the obese population; however, it is worth noting that the antiproteinuria as well as renoprotective effects of angiotensin-converting enzyme (ACE) inhibition were greater in obese than in nonobese patients [108]. Ox-LDL accumulation in the glomeruli stimulates downstream RAS-mitogen activated protein kinase (MAP kinase) signaling cascade leading to mesangial cell proliferation [109]. RAAS activation mediates fatty acid-induced endoplasmic reticulum stress in cultured human proximal tubule cells (HK2) and in mice fed with a high-fat diet [110]. Treatment with the angiotensin II type 1 receptor blocker valsartan, or renin inhibitor aliskiren, significantly suppressed ER stress both in vitro and in vivo [110, 111].

Peroxisome proliferator-activated receptor-alpha (PPAR-*α*) is a transcription factor predominantly expressed in metabolically active tissues, such as renal PTC, and regulates FAO. Lipid accumulation due to FAO inhibition indirectly contributes to fibrogenesis by accelerating inflammation. Researchers using animal and cell models have revealed that agonists of PPAR-*α* showed benefits in reversing defects in FAO and ameliorating CKD progression [112]. The PPAR-*α*/PPAR-*γ* coactivator-1a (PPARGC1A) ensemble plays a dominating role in the regulation of FAO, which is an available therapeutic target in the future. Fenofibrate (a PPAR-*α* agonist) could enhance FAO in the kidneys and has shown a positive effect in mouse models of CKD [113]. Fenofibrate could reduce renal oxidative stress, systemic triglyceride levels, proteinuria, and glomerulosclerosis, thereby comprehensively improving renal function in mice fed HFD [114]. Cholesterol efflux through the PPAR-liver X receptor alpha-ABCA1 pathway is damaged in IL-1*β*-treated mesangial cells; however, such a phenomenon can be reversed by PPAR-*α* agonists by the activation of the “ABCA1 cholesterol efflux” pathway, producing mesangial cells free of IL-1*β*-governed intracellular lipid accumulation [113]. The overexpression of proximal tubular epithelial cell-specific PPAR-*α* in mice sufficiently maintained FAO and conferred protection against ischemia/reperfusion injury (IRI) [115]. Multiple clinical trials have also demonstrated the reduction in albuminuria in patients with diabetes when given fenofibrate treatment [4, 116]. For therapeutic purposes, agonists of PPAR-*α* were widely applied to impede cisplatin-induced acute kidney injury (AKI), ischemia/reperfusion injury (IRI), and FFA accumulation [117–119]. These observations validated the potential therapeutic uses of PPAR-*α* agonists [3]. It has been reported that PPAR-*γ* agonists might also protect against renal injury via their antifibrotic and anti-inflammatory effects [120–122].

The farnesoid X receptor (FXR) is another potent therapeutic target that is highly expressed in the kidney [123]. In mice fed HFD and in age-related kidney disease models, increased renal expression of SREBP-1 plays a key role in kidney lipid accumulation and increases the activity of proinflammatory cytokines [106]. Mice treated with an FXR-activating ligand had lower accumulation of triglycerides by regulating fatty acid synthesis and oxidation, which is related to reduced proteinuria and prevents the loss of podocytes [124, 125]. Studies have shown that in HFD-induced obese mice, an FXR agonist protects the kidneys by downregulating the expression level of SREBP-1 [126]. The treatment of obese mice with the semisynthetic FXR agonist obeticholic acid (OCA) reduced the degree of glomerular sclerosis and tubulointerstitial injury by improving mitochondrial function and promoting FA oxidation, which, in turn, reduced mitochondrial stress and ER stress [126]. In another study, administration of OCA to mice prevented early-stage renal damage and protected the kidney from CKD in the long term [123]. In addition, a recent study on nonalcoholic fatty liver disease has shown that the anti-inflammatory effect of FXR activation was the result of a switch in arachidonic acid metabolism [126]. Pharmacological activation of FXR appears to be safe and represents a valid treatment option for the continuously increasing number of overweight patients with CKD [117, 123].

Similar results were observed for doxercalciferol, which is a typical vitamin D agonist [127]. Pioglitazone also has beneficial effects on albuminuria diabetic and obese patients [68].

## 6. A Vicious Circle of Lipid Disorders and Kidney Disease

Dyslipidemia accelerates the progression of CKD and subsequently causes secondary abnormalities, in particular, in lipid metabolism [3]. As an example, diabetes-induced hypertriglyceridemia results from multiple processes, including enhanced triglyceride generation, faster de novo synthesis stimulated by hyperinsulinemia in type 2 diabetes mellitus (T2DM), and the defective removal of plasma triglyceride [128]. These lipid abnormalities are closely correlated with the preserved kidney function prevailingly in diabetic patients. Notably, nondiabetic patients with CKD have similar symptoms of dyslipidemia [129, 130]. CKD also leads to a decrease in FAO, which might be another mechanism resulting in lipid accumulation. The defective utilization of fatty acids leads to energy consumption causing apoptosis and dedifferentiation, eventually contributing to renal fibrosis and CKD progression [4].

## 7. Summary

The aim of this paper was to review recent progress in the understanding and uncovering of the micromechanisms of CKD and lipid abnormalities. The key hypothesis we believe is that lipid abnormalities directly cause CKD and thus constitute a vicious cycle. Such a seemingly abnormal relationship was theoretically and experimentally validated by elaborately elucidating lipid intake and accumulation as well as their influence on CKD. For all the phenomena considered, it has hopefully been clearly demonstrated that feasible treatment options and great efforts will contribute to advances in the technological and scientific knowledge required to more efficiently prevent and treat CKD.
